# Thymoquinone ameliorates some endocrine parameters and histological alteration in a rat model of polycystic ovary syndrome

**Published:** 2018-04

**Authors:** Sima Taghvaee Javanshir, Parichehreh Yaghmaei, Zahra Hajebrahimi

**Affiliations:** 1 *Department of Biology, Science and Research Branch, Islamic Azad University, Tehran, Iran. *; 2 *Aerospace Research Institute, Ministry of Science, Research and Technology, Tehran, Iran.*; * Parichehreh Yaghmaei and Zahra Hajebrahimi are an equal Corresponding Author.

**Keywords:** Estradiol valerate, Polycystic ovary syndrome, Rat, Thymoquinone

## Abstract

**Background:**

Polycystic ovary syndrome (PCOS) is a common form of the endocrine disease which is associated with metabolic dysfunction. PCOS and type 2 diabetes mellitus are related in multiple aspects and are similar in many pathological features. Anti-diabetic effects of *Nigella sativa* and protective effects of it on reproductive system have been suggested in some reports.

**Objective:**

The aim of current study was to evaluate the effects of thymoquinone, the main components of *Nigella sativa*, on PCOS model of rats.

**Materials and Methods:**

Intraperitoneal injection of estradiol valerate for 25 days was used to induce PCOS in Wistar rats, followed by intraperitoneal administration of 8 and 16 mg/kg thymoquinone for 30 days. Rats were divided into 5 groups; control, sham or PCOS, experiment-1 (PCOS and 8 mg/kg thymoquinone), experiment-2 (PCOS and 16 mg/kg thymoquinone), and metformin (PCOS and metformin administration, 100 mg/kg) groups. All of the animals were subjected to serum biochemical analysis of blood and histopathological study of ovaries.

**Results:**

Estradiol valerate induced PCOS while administration of thymoquinone recovered it. The body weight, ovarian morphology, and ovulation had been improved and the serum biochemical parameters including glucose, triglyceride, total cholesterol, low-density lipoprotein, high-density lipoprotein, luteinizing hormone, and follicle stimulating hormone were reversed after thymoquinone intervention.

**Conclusion:**

Our data suggest that thymoquinone has improvement effects on an ovarian function and ovulation in the PCOS rat model. Therefore, thymoquinone and *Nagilla sativa* could be used as a protective agent and as an adjunct treatment in PCOS patients.

## Introduction

Polycystic ovary syndrome (PCOS) is the most common form of hormonal imbalance among young women (1). Women with PCOS suffer from anovulation, hyperandrogenism, irregular or no menstrual periods, and fertility problems. It increases the risk of hyperinsulinemia, type 2 diabetes mellitus, cardiovascular dysfunction, endometrial cancer and mental health impairment (2).

Metabolic dysfunction in women with PCOS is very common. These women often have higher levels of low-density lipoprotein (LDL), triglycerides (TG), cholesterol, and low level of high-density lipoprotein (HDL) in their blood serum that may lead to the heart disease (3). Also, PCOS is characterized by elevated luteinizing hormone (LH) and decreased follicle stimulating hormone (FSH) levels (increased LH: FSH ratio). In fact, LH may increase in response to elevated androgens that can develop anovulation and infertility later (4-6). Therefore, one strategy for PCOS therapy is regulating the imbalance levels of hormones and using medication that helps to improve insulin resistance. 

Few chemical agents have been approved by the U.S. Food and Drug Administration for use in PCOS including Metformin (7). Metformin was the first insulin sensitizing drug to be used in PCOS. It has positive effects on anovulation, insulin resistance and obesity features of PCOS. Also, it has demonstrated that metformin supports ovarian function, increase the rate of pregnancy and improve the infertility problems (8, 9). 

From past to present, natural products have played an important role in the development of new sources to treat disease. In recent years, using the alternative medicine, especially herbal medicine has become popular in both developing and developed countries due to their accessibility, no or fewer side effects and ease of use (10). *Nigella sativa* (*N. sativa*), has been used for the treatment of various diseases throughout the world. It is an annual herbaceous plant from the Ranunculaceae family, native to Mediterranean regions (11). Many therapeutic properties of *N. sativa* have been showed including anti-inflammatory, anti-oxidative, anti-tumor and neuroprotective effects (12-14). 

It was proved that the extract of *N. sativa* has a hypoglycemic effect on type 2 diabetes mellitus (15, 16). Besides anti-diabetic activity, recent studies have shown that *N. sativa* has the positive effect on the reproductive system, too (17). 

In addition, study by Kamarzaman and co-worker demonstrated the prophylactic effect of *N. sativa* on the number of ovarian follicles and diameters against cyclophosphamide in adult mice (18). With respect to the above reports, the present work aims to investigate the effects of thymoquinone (one of the compounds of *N. sativa*) on prevention and reducing symptoms related to PCOS.

## Materials and methods

**Experimental animals and estradiol valerate treatment**

The present work is an experimental study. Thirty adult female Wistar rats (5 wk old) weighing between 180-200 gr, were purchased from Razi laboratory animal, Islamic Azad University, Science and Research Branch, Tehran, Iran. 

Animals were kept in the animal house of Science and Research Branch of Islamic Azad University, under standard laboratory conditions of constant temperature (22^o^C) and humidity with the 12 hr light/dark cycle. All efforts were made to minimize animal suffering and to reduce the number of animals used. All animals were weighed every week and changes in body weight were recorded during the experimental period. PCOS was induced by an intraperitoneal injection of 4 mg estradiol valerate (Aburaihan Pharmaceutical Co; Tehran, Iran) for 25 days and demonstrated through histological analyzing of ovaries. 

Animals were randomly divided into five groups (n=6/each): 1- control group; 2- sham operation group: PCOS rats which received intraperitoneal injection of tween (Sigma, USA) (80%) as thymoquinone solvent for 30 days; 3- experimental group 1 (EXP-1): PCOS rats treated with thymoquinone (8 mg/kg, Sigma Chemical Co, St. Louis, MO, USA) for 30 days; 4- experimental group 2 (EXP-2): PCOS rats treated with thymoquinone (16 mg/kg) for 30 days; and 5-metformin group: PCOS rats with metformin administration (100 mg/kg, Osve Pharmaceutical Co, Iran) for 30 days.

**Blood Sampling and biochemical analysis**

Serum samples and ovary tissues were collected at the end of treatment period. For this purpose, rats were anesthetized with ether and blood samples were obtained from the heart with a syringe and left at room temperature for 2 hr. Serum was separated by centrifugation at 2500×g for 5 min and immediately stored at -20^o^C until analysis. Concentration of total cholesterol (TC), HDL, LDL, and TG were measured according to the methods described by Loeffler and McDougald (19), Burstein and co-worker (20), Friedewald and colleagues (21), and Gottfried and Rosenberg (22), respectively.

The concentration of these parameters was estimated by animal biochemical kits purchased from Ziest Chem Diagnostic Company (Tehran, Iran) according to the manufacturer’s recommendations. Serum glucose level was estimated by an animal enzymatic colorimetric glucose oxidase assay kit (Ziest Chem Diagnostic Company; Tehran, Iran). Also, blood glucose meter (Cera pet, South Korea) was used to measure and display the amount of whole blood sugar every week in blood obtained from the animal tail.

Serum FSH and LH level were measured using rodent FSH and rodent LH ELISA test kits (Cosmo Bio Co. Ltd. Japan) that are immunoassay designed for the quantitative determination of LH and FSH in serum samples of rodents. The assay system utilizes a polyclonal anti-LH / anti-FSH antibody for solid phase immobilization and mouse anti-LH/ anti-FSH antibody in the antibody-enzyme (horseradish peroxidase) conjugate solution.

**Histological procedure**

Ovaries were removed and evaluated for histological analysis at the end of treatment. Samples were cut, trimmed free of fat, fixed in 10% paraformaldehyde for 24 hr and then embedded in paraffin after standard processing of dehydration and clearing. Then paraffin samples were sectioned at a thickness of 6 μm, mounted on glass slides, stained with Hematoxylin and Eosin and observed under a light microscope (23). Primary, secondary (or antral follicle) and Graafian follicles were counted in all samples according to the Erickson’s classification (24). Only follicles with a visible nucleus were enumerated. Corpus luteum and follicular cysts were estimated, too. Also, the thickness of antral follicles theca layer and granulosa layer and follicular diameters were measured.

**Ethical consideration**

Animal had unlimited access to food and water. They were cared for in accordance with the Guide for the Care and Use of Laboratory Animals (25). All protocols described here were approved by the Animal Care and Use Committee of Islamic Azad University, Science and Research Branch, Tehran, Iran (Code:IR.IAU.SRB.REC.1396.11).

**Statistical analysis**

SPSS statistical software (Statistical Package for the Social Sciences, version 17.0, SPSS Inc, Chicago, Illinois, USA) was used for data analyzing. All data are presented as the means±S.E.M. One-Way ANOVA (one-way analysis of variance) with Tukey test was used for analyzing the data and comparison of the different group means. p≤0.05 was considered as significant. 

## Results

**Measurement of weight and biochemical assessments of blood**

Table I summarizes the body weight in all animal groups were taken during the 8 wk of the experimental period (25 days for injection of estradiol valerate to induce PCOS and 30 days for administration of thymoquinone or metformin). Intraperitoneal injection of estradiol valerate resulted in a reduction in the body weight of all animals in comparison to control group. The mean starting weight for the control group was 203±1.41 at the 1^st^ wk. The weight loss continued throughout the rest of the experiments (until wk 8) in the sham operation group (p=0.001) compared to the control group. In contrast to sham operation group, administration of thymoquinone or metformin compensated weight loss. Our data showed a significant increase (p=0.01) in body weight in the metformin group and EXP-1 and EXP2 by the end of the treatment period (8 wk) in comparison to sham operation group. 

The blood glucose levels of all groups during the experimental weeks are presented in table II. Our data revealed a marked increase in the level of blood glucose in all groups after the induction of PCOS compared to the control group. This increase remained throughout the rest of the trials in the sham operation group (p=0.001). Administration of thymoquinone or metformin significantly decreased the blood glucose level of the EXP-1, EXP-2 and metformin groups and its amount came back to the control levels by the end of the experiments (p=0.01).

The serum levels of glucose, TG, TC, LDL, HDL, FSH, and LH are presented in table III. We observed that changes in serum glucose were aligned with changes in blood sugar. The obtained data showed hyperglycemia in sham operation group but treatment with thymoquinone or metformin improved it (p=0.01). As shown in table III, injection of estradiol valerate decreased the serum level of HDL in sham animals when compared with control rats. As mentioned for glucose, administration of thymoquinone or metformin, especially thymoquinone at the concentration of 16 mg/kg (EXP-2 group), enhanced the value of HDL in serum. In contrast to HDL, induction of PCOS significantly increased the content of TC, TG and LDL in sham rats (p=0.001). However, their levels decreased to normal or near normal values following thymoquinone or metformin treatment in EXP-1, Exp-2 and metformin group in comparison to sham ones. With respect to our result, changes in serum levels of FSH and LH hormones were similar to HDL and LDL changes, respectively.

**Ovarian morphology**

**Figure 1:**
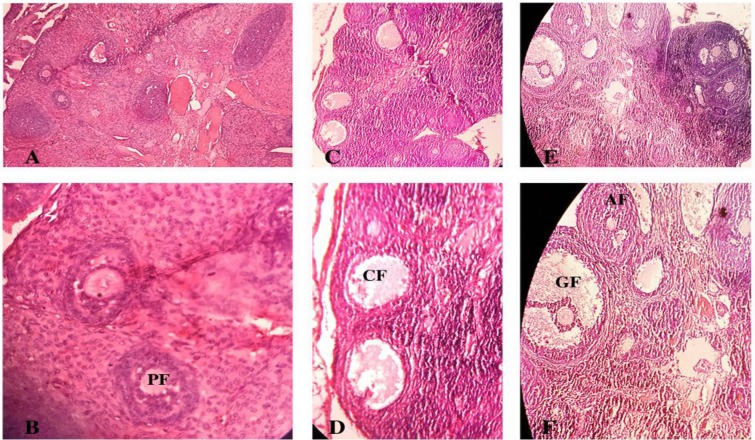
Photomicrograph of a section in ovarian tissues of adult control (A, B), sham (C, D) and EXP-2 (E, F) rats using hematoxylin and eosin staining showing primary follicle (PF), the cystic follicle (CF), graafian follicle (GF), and antral follicle (AF). (A, C, and E: 40X; B and D: 400X; F: 100X

**Figure 2:**
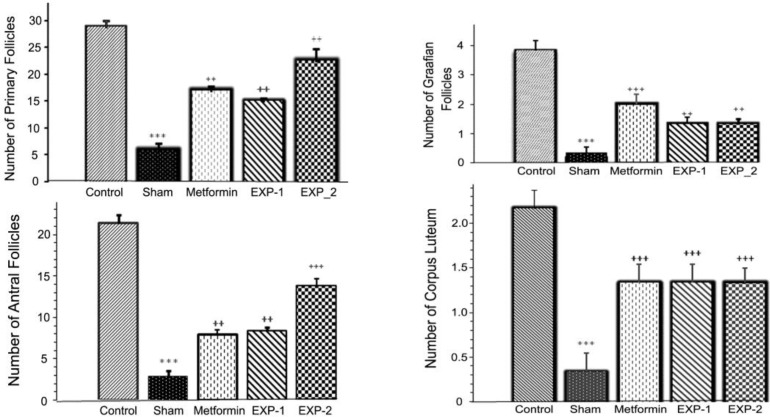
Number of primary follicles, antral follicles, graafian follicles, and corpus luteums in control, sham, metformin, EXP-1, and EXP-2 rats after 8 wk of experimental period. Values are presented as mean±SE. (n=6/each group). *** Statistically different from the control rats (p≤0.001), ++ statistically different from the sham rats (p≤0.01), +++ statistically different from the sham rats (p≤0.001

The ovarian sections taken from the control animals showed a natural structure with follicles at different stages of growth and normal granulosa cell layers (Figure 1 A, B). Also, a large number of corpus luteum was observed that indicated ovulation. On the contrary, the ovary of estradiol valerate induced PCOS rats (sham operation group), showed multiple numbers of large fluid-filled cystic follicles with a reduced and abnormal granulosa cells (Figure 1 C, D). In comparison with control animals, the numbers of primary follicles, antral follicles, Graafian follicles and corpus luteum (Figure 2) significantly decreased (p=0.001) in sham operation group. 

**Figure 3.**
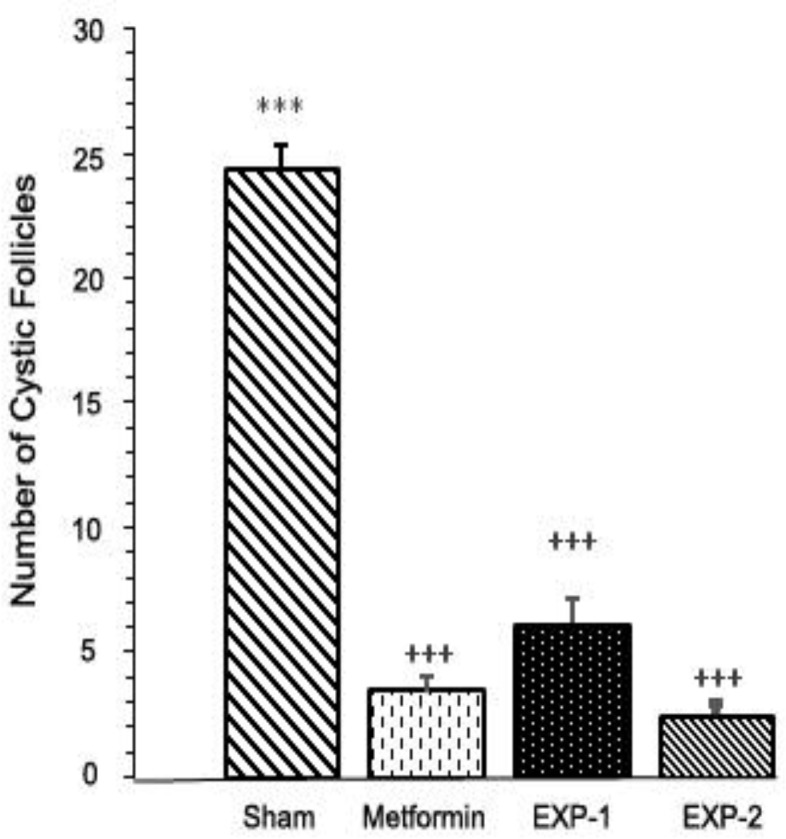
Number of cystic follicles in sham, metformin, EXP-1, and EXP-2 rats after 8 wk of the experimental period. Values are presented as mean±SE. (n=6/each group). *** Statistically different from the control rats (p≤0.001), +++ statistically different from the sham rats (p≤0.001

In comparison with the ovaries of sham rats, treatment with thymoquinone (8 or 16 mg/kg) or metformin for 30 days, significantly decreased the number of follicular cysts (Figure 3) in EXP-1, EXP-2 and metformin groups (p=0.001). Furthermore, injections of thymoquinone increased considerably the number of primary follicles, antral follicles (p=0.001), Graafian follicles (p=0.01) and corpus luteum (p=0.001) in treated rats (Figure 2). Similar effects were found in animals that treated with metformin. The results of morphometry analysis are presented in table IV. 

The size of the follicles and the thickness of the theca layer significantly increased in sham operation group (p=0.05) compared to the control group. In comparison with sham animals, the diameter of follicles and the theca layer thickness significantly decreased (p=0.05) following administration of Metformin and thymoquinone in Metformin, Exp-1, and EXP-2 groups. However, a significant decrease was obtained in the thickness of granulosa layer between sham group and the control group (p=0.05). Metformin and thymoquinone increased the thickness of the granulosa layer in Metformin, Exp-1, and EXP-2 groups (p=0.05).

**Table I:** Body weight (gr) of the rats during the 8 wk

**Group**	**Week 1**	**Week 2**	**Week 3**	**Week 4**	**Week 5**	**Week 6**	**Week 7**	**Week 8**
Control	203 ± 1.41	205 ± 2.55	208 ± 0.81	213 ± 2.15	215 ± 1.41	216 ± 2.23	218 ± 2.70	220 ± 2.16
Sham	190 ± 2.15	180 ± 0.81**	173 ± 2.55***	167 ± 1.63***	163 ± 2.70**	160 ± 3.3***	159 ± 1.28***	157 ± 2.03***
EXP-1	196 ± 2.15	188 ± 2.44**	176 ± 0.88***	167 ± 2.08***	163 ± 2.16	164 ± 1.63	163 ± 0.81	176 ± 1.15++
EXP-2	197 ± 2.94	189 ± 1.41**	175 ± 1.82***	168 ± 0.41***	165 ± 1.28	165 ± 1.82	166 ± 1.41	175 ± 1.63++
Metformin	188 ± 0.81	180 ± 2.08**	172 ± 1.48***	167 ± 1.28***	164 ± 1.41	163 ± 1.63	164 ± 2.1	173 ± 2.44++

Values are presented as mean±SE (n=6/each group).

Sham: PCOS rats + i.p. injection of tween (80%)

EXP-1: PCOS rats + i.p. injection of thymoquinone (8 mg/kg)

EXP-2: PCOS rats + i.p. injection of thymoquinone (16 mg/kg)

** Statistically different from the control rats (p≤0.01),

++ statistically different from the sham rats (p≤0.01),

*** statistically different from the control rats (p≤0.001).

**Table II:** The blood glucose level (mg/dl) of rats during the 8 wk

**Group**	**Week 1**	**Week 2**	**Week 3**	**Week 4**	**Week 5**	**Week 6**	**Week 7**	**Week 8**
Control	85 ± 2.70	83 ± 1.41	80 ± 1.28	82 ± 0.81	81 ± 0.81	84 ± 1.28	80 ± 1.28	84 ± 1.28
Sham	101 ± 1.63**	102 ± 2.16**	110 ± 2.94***	116 ± 2.22***	121 ± 2.51***	121 ± 2.44***	122 ± 2.15***	122 ± 2.1***
EXP-1	100 ± 1.82**	105 ± 2.44**	108 ± 1.63***	115 ± 2.08***	108 ± 0.81+	98 ± 1.72++	92 ± 1.82++	88 ± 1.28++
EXP-2	103 ± 2.70**	106 ± 1.41**	114 ± 0.41***	117 ± 0.81***	110 ± 0.81+	101 ± 1.28++	89 ± 1.41++	84 ± 1.28++
Metformin	99 ± 1.41**	103 ± 2.08**	112 ± 2.39***	116 ± 1.63***	106 ± 0.81+	100 ± 1.82++	90 ± 1.63++	85±0.81++

Values are presented as mean ± SE (n=6/each group).

Sham: PCOS rats + i.p. injection of tween (80)

EXP-1: PCOS rats + i.p. injection of thymoquinone (8 mg/kg)

EXP-2: PCOS rats + i.p. injection of thymoquinone (16 mg/kg)

** Statistically different from the control rats (p≤0.01),

*** statistically different from the control rats (p≤0.001),

+ statistically different from the sham rats (p≤0.05),

++ statistically different from the sham rats (p≤0.01).

**Table III:** Biochemical findings of the PCOS rats and controls

**Group**	**Glucose (mg/dl )**	**TG (mg/dl )**	**TC (mg/dl )**	**LDL (mg/dl )**	**HDL (mg/dl )**	**FSH (u/l )**	**LH (u/l )**
Control	121 ± 2.08	96 ± 0.2	83 ± 1.12	17 ± 1.5	35 ± 0.81	2.3 ± 0.05	0.38 ± 0.015
Sham	158 ± 2.08***	129 ± 0.4***	98 ± 1.76**	38 ± 1.15***	20 ± 1.15***	1.06 ± 0.04***	0.74 ± 0.03***
EXP-1	122 ± 2.18++	126 ± 1.5+	85 ± 2.0++	26 ± 1.15++	26 ± 2.0++	1.57 ± 0.22++	0.52 ± 0.02++
EXP-2	124 ± 1.45++	118 ± 1.8++	83 ± 2.4++	16 ± 1.45+++	38 ± 1.4+++	1.64 ± 0.18++	0.55 ± 0.02++
Metformin	125 ± 2.5++	120 ± 0.7++	84 ± 2.5++	22 ± 1.76++	29 ± 2.7++	1.75 ± 0.16++	0.51 ± 0.06++

Values are presented as mean±SE. (n=6/each group).

Sham: PCOS rats + i.p. injection of tween (80)

EXP-1: PCOS rats + i.p. injection of thymoquinone (8 mg/kg)

EXP-2: PCOS rats + i.p. injection of thymoquinone (16 mg/kg)

TG: Triglyceride

TC: Total cholesterol

LDL: Low-density lipoprotein

HDL: High-density lipoprotein

FSH: Follicle stimulating hormone

LH: Luteinizing hormone

** Statistically different from the control rats (p≤0.01),

*** statistically different from the control rats (p≤0.001),

+ statistically different from the sham rats (p≤0.05),

++ statistically different from the sham rats (p≤0.01),

+++ statistically different from the sham rats (p≤0.001).

**Table IV:** Morphometry findings of the PCOS rats and controls is correct

**Group**	**Follicle diameter (µm)**	**Theca layer thickness (µm)**	**Granulosa layer thickness (µm) **
Control	350 ± 1.29	26 ± 0.95	59 ± 1.1
Sham	440 ± 0.81*	44 ± 0.83*	32 ± 0.94*
EXP-1	360 ± 1.18+	34 ± 0.86+	49 ± 0.97+
EXP-2	355 ± 1.32+	31 ± 0.96+	52 ± 0.86+
Metformin	353 ± 0.79+	29 ± 2.06+	53 ± 1.7+

Values are presented as mean±SE (n=6/each group).

Sham: PCOS rats + i.p. injection of tween (80)

EXP-1: PCOS rats + i.p. injection of thymoquinone (8 mg/kg)

EXP-2: PCOS rats + i.p. injection of thymoquinone (16 mg/kg)

TG: Triglyceride

TC: Total cholesterol

LDL: Low-density lipoprotein

HDL: High-density lipoprotein

FSH: Follicle stimulating hormone

LH: Luteinizing hormone

* Statistically different from the control rats (p≤0.05),

+ statistically different from the sham rats (p≤0.05)

## Discussion

In the present study, Intraperitoneal injection of estradiol valerate was used for the experimental developing model of PCOS rats during 25 days. Administration of estradiol valerate has been proved to induce laboratory model of PCOS in rodents (27). It can induce metabolic dysfunction, hormonal alterations, anovulation, and morphological changes and follicular cystic in ovaries. PCOS is associated with increases in LH levels and decreases in FSH levels, which we also found in sham rats. These results indicated that the PCOS model was successfully established. We also observed metabolic dysfunction including dysglycemia and dyslipidemia. 

Our data indicated that 25 days injection of estradiol valerate significantly increased the serum and blood levels of glucose in sham animals. They also showed cholesterol and lipid abnormalities, such as raised serum levels of TC, TG, and LDL and reduced levels of HDL. The histological finding also confirmed the induction of PCOS in the sham group. Multiple numbers of cystic follicles with a reduced or abnormal granulosa cells were observed in the ovaries of sham rats. There were also smaller numbers of primary follicles, antral follicles, Graafian follicles and corpus luteum, which suggests anovulation. These findings again demonstrate that injection of estradiol valerate has been able to create PCOS models in the rat. 

PCOS is often associated with overweight. Obesity is one of the risk factors that can worsen other conditions of PCOS (1, 2). Our data showed that PCOS induction by estradiol valerate resulted in a reduction in the body weight of all animals when compared with control group. These results confirmed previous data reported by Stener-Victorin and co-worker (28). They suggested that injection of estradiol valerate increased the metabolism of lipids which reduced the body weight of animals. However, all the women with PCOS don’t show obesity symptom. Besides, studies have shown that PCOS enhances the function of the sympathetic system (29). This also increases lipids metabolism which consequently leads to body weight loss (29).

To date, the only known effective medicine for PCOS is metformin (30). In fact, metformin is a first line drug that used for the treatment of type 2 diabetes mellitus alone or in combination with other medications to improve blood sugar control (8). It is an insulin sensitizing drug that reduces the body weight and has an improving effect on ovulation and fertility (31). It also helps to improve symptoms of hyperinsulinemia through decreasing hepatic gluconeogenesis, increasing insulin sensitivity of different tissues for glucose uptake (32), and reducing serum levels of elevated androgen (33). It has been showed that metformin has positive effects on oocyte maturation, too (34). 

Our data confirmed previous studies. We showed that metformin improved adverse features induced by estradiol valerate in the metformin group. It is improved both metabolic dysfunction and hormonal changes in metformin rats. We observed that metformin decreased the serum levels of glucose, TG, total cholesterol, LDL and LH hormone in metformin group in comparison to sham animals. In contrast, administration of metformin increased the serum levels of HDL and FSH hormone. Histological analysis also confirmed biochemical findings. We found that administration of metformin improved the numbers of primary follicles, antral follicles, Graafian follicles and corpus luteum in treated rats. In addition, we observed that the numbers of follicular cystic considerably decreased in the metformin group. 

Here we investigated the therapeutic effects of thymoquinone in a rat model of PCOS disease. In this present work, thymoquinone at a dose of 8 and 16 mg/kg was used (EXP-1 and EXP-2 groups, respectively). Similar results with metformin were found in animals that treated with thymoquinone. According to our results, administration of thymoquinone could improve symptoms of PCOS and restore normal ovulation in the PCOS Wistar rat model in a similar way to metformin. 

Thymoquinone is major bioactive components of *Nigella sativa* which have potential therapeutic properties. Many therapeutic properties of *Nigella sativa* have been showed including anti-inflammatory, anti-oxidative, anti-diabetic, anti-tumor and neuroprotective effects (12-14). Polycystic ovary syndrome exhibits chronic inflammatory behavior. Therefore, therapeutic effects of thymoquinone on PCOS may be due to its anti-inflammatory properties.

Due to the linkage between PCOS and type 2 diabetes mellitus, we decided to examine the effects of thymoquinone in a PCOS rat model. In the present study, administration of thymoquinone (8 mg/kg) decreased the level of blood glucose in EXP-1 group in comparison to the sham group. Increasing the level of thymoquinone (16 mg/kg) caused further decreasing of blood glucose (Table II). The amount of blood glucose came back to the control levels in the EXP-2 group after completion of treatment. This result confirmed data reported previously by others. Previous studies by Al-Hader *et al* and El-dien and co-worker were proved that the extract of *N. sativa* has the hypoglycemic effect on type 2 diabetes mellitus (15, 16). 

Al-Hader and colleagues showed that intraperitoneal injection of extract from *Nigella sativa* seeds to normal and diabetic rabbits caused considerable reduction in the fasting glucose levels. In another study, El-dien and co-worker reported that extract of *N. sativa* had anti-diabetic effects on diabetic adult male rats. Some studies revealed that the extract of *N. sativa* seeds increases glucose-induced insulin release in isolated Langerhans islets from rats (16). Some other studies also have suggested anti-diabetic effects of *N. sativa* and thymoquinone (35, 36).

Type 2 diabetes mellitus is associated with dislipidemia including a high level of triglyceride, total cholesterol and low-density lipoprotein and a low level of high-density lipoprotein which enhances the risk for heart diseases and stroke. Kaatabi and co-worker have shown that administration of *N. sativa* seeds at a dose of 2 gram per day for 12 wk in patients with type 2 diabetes mellitus could improve the dyslipidemia features of the disease (37). They observed a considerable decrease in triglyceride, total cholesterol, low-density lipoprotein and a considerable elevation in high-density lipoprotein in patients with type 2 diabetes mellitus after treatment with *N. sativa* seeds. Similar results were obtained in our study. 

Our data revealed that after administration of thymoquinone, there were significant differences between the sham group and experimental group number 1 and 2 in serum levels of TG, TC, LDL, and HDL. Administration of thymoquinone significantly decreased the serum levels of TG, TC, and LDL and increased HDL level both in EXP-1 and EXP-2 group in comparison to sham animals. These results again confirm the anti-diabetic effects of *N. sativa* and due to the linkage between PCOS and type 2 diabetes mellitus; it could prescribe for PCOS, too. The serum levels of TC and HDL were no significant between the EXP-1 and EXP-2 groups. In contrast, there were obvious differences in serum levels of TG and LDL between the two groups, suggesting an improvement in TG and LDL concentration in a dose-dependent manner.

Besides anti-diabetic activity, recent studies have shown that *N. sativa* has the positive effect on the reproductive system, too. Enhancing effects of *N. sativa* on fertility potential, the level of LH and testosterone hormone in male rats has been established (17). Mukhallad and co-worker and Al-Sa’aidi and co-worker have shown that administration of *N. sativa* increased the sperm motility, spermatogenesis and the number of impregnated female rats by males receiving treatment (38, 39). In addition, a recent study by Kamarzaman and co-worker demonstrated the prophylactic effect of *Nigella sativa* on the number of ovarian follicles and diameters against cyclophosphamide in adult mice (18).

Our data confirmed positive effects of thymoquinone on the reproductive system, too. Treatment with thymoquinone (8 or 16 mg/kg) significantly decreased the number of follicular cysts in EXP-1 and EXP-2 groups. Furthermore, injections of thymoquinone increased considerably the number of primary follicles, antral follicles, Graafian follicles and corpus luteum in treated rats. The number of follicular cysts, primary follicles, Graafian follicles and corpus luteum was no significant between the EXP-1 and EXP-2 groups. 

## Conclusion

In conclusion, our data in correlation with histological and biochemical analysis revealed that Intraperitoneal injection of estradiol valerate could be induced PCOS in Wistar rats while administration of thymoquinone could prevent adverse features of the disease. Our data also confirmed anti-diabetic effects of thymoquinone and suggested protective effects on an ovarian function and ovulation in the PCOS rat model. Therefore, thymoquinone and *Nigella sativa* may be used as an adjunct treatment in PCOS patients. 

## Note

***This article extracted from M.Sc. Thesis. (Sima Taghvaee Javanshir)***
